# A Mindfulness-Based Brain-Computer Interface to Augment Mandala Coloring for Depression: Protocol for a Single-Case Experimental Design

**DOI:** 10.2196/20819

**Published:** 2021-01-18

**Authors:** Claudia Daudén Roquet, Corina Sas

**Affiliations:** 1 School of Computing and Communications Lancaster University Lancaster United Kingdom

**Keywords:** brain-computer interface, mental well-being, depression, mindfulness, mandala coloring

## Abstract

**Background:**

The regular practice of mindfulness has been shown to provide benefits for mental well-being and prevent depression relapse. Technology-mediated interventions can facilitate the uptake and sustained practice of mindfulness, yet the evaluation of interactive systems, such as brain-computer interfaces, has been little explored.

**Objective:**

The objective of this paper is to present an interactive mindfulness-based technology to improve mental well-being in people who have experienced depression. The system, Anima, is a brain-computer interface that augments mandala coloring by providing a generative color palette based on the unfolding mindfulness states during the practice. In addition, this paper outlines a multiple-baseline, single-case experimental design methodology to evaluate training effectiveness.

**Methods:**

Adult participants who have experienced depression in the past, have finished treatment within the last year, and can provide informed consent will be able to be recruited. The Anima system, consisting of 2 tablets and a nonintrusive mental activity headband, will be delivered to participants to use during the study. Measures include state and trait mindfulness, depression symptoms, mental well-being, and user experience, and these measures will be taken throughout the baseline, intervention, and monitoring phases. The data collection will take place in the form of a questionnaire before and after each mandala-coloring session and a semistructured interview every 2 weeks. Trial results will be analyzed using structured visual analysis, supplemented with statistical analysis appropriate to single-case methodology.

**Results:**

Study results will offer new insights into the deployment and evaluation of novel interactive brain-computer interfaces for mindfulness training in the context of mental health. Moreover, findings will validate the effectiveness of this training protocol to improve the mental well-being of people who have had depression. Participants will be recruited locally through the National Health Service.

**Conclusions:**

Evidence will assist in the design and evaluation of brain-computer interfaces and mindfulness technologies for mental well-being and the necessary services to support people who have experienced depression.

**International Registered Report Identifier (IRRID):**

PRR1-10.2196/20819

## Introduction

### Background

Recent developments in interventions for depression and the prevention of its relapse have focused on applying mindfulness-based strategies, which aim to shift the focus of attention away from the negative content of thought to nonjudgmentally observe the mind processes [1-3]. It has been long suggested that recovered patients with depression should be trained in mindful self-awareness to counter rumination and reduce the risk of future relapses [4]. Mindfulness, with its origins in Buddhist traditions, has been defined in Western psychology as a process of deliberate, nonjudgmental self-regulation of attention to the present moment-to-moment experience without being distracted by thoughts of the past or future [5]. Landmark examples of psychology programs that aim to improve mental and physical health are the mindfulness-based stress reduction [6], mindfulness-based cognitive therapy [7], and mindfulness-based art therapy (MBAT) [8] programs. The underlying mechanisms of mindfulness training have also been widely investigated, and there is an agreement in the literature that mindfulness is a metacognitive attentional process that is concerned with how individuals relate to the content of their thoughts [9]. This specific relational process is believed to reduce the ruminative aspects of depression by altering the way individuals view their own process of thought [4,10,11].

MBAT is based on the self-regulation theory and integrates mindfulness skills and aspects of art therapy into an 8-week, gender-segregated, supportive group therapy format [12,13]. The overall goal is to provide specific skills for cultivating self-regulation of attention and affect in a format that is not confined to verbal processing alone. It provides a foundation for understanding reactions to perceptions of physical and emotional well-being. A common activity in MBAT is the coloring of mandalas for self-awareness, self-expression, conflict resolution, and healing [14]. Mandalas, originally from Tibetan Buddhism, were introduced into psychotherapy by Carl Jung [15]. He suggested that the act of drawing mandalas had a calming and healing effect on its creator, while simultaneously facilitating psychic integration and personal meaning in life [16]. The mandala functions as a symbolic representation of emotionally laden and conflicting material, yet at the same time provides a sense of order and integration to this material [17-23].

Our research draws from previous scientific investigation of the benefits of mandala coloring for mental health [24] and previous studies we conducted exploring this practice and its impact on well-being with the general population [25,26]. The approach presented in this paper differs from previous MBAT programs, as it is an individual self-care approach that uses interactive technology, which we named Anima, to decrease depressive symptoms and increase mental well-being in people who have experienced depression in the past. Anima is a mindfulness-based technology that was designed and developed after an exploratory study with experts on the practice of mandalas, in which we found that people used mandalas as a self-care tool for their mental well-being (paper submitted for publication). Experts described how the coloring of the mandala allowed for the expression of affective and mental states that would otherwise be difficult to communicate. We found that the colors used during coloring were used to express such underlying emotions, and they served as emotional cues in the final mandala to facilitate reflection on their experiences. Therefore, mandala coloring seemed to support both attention and emotion regulation strategies [27]. Furthermore, the coloring process of the mandala was seen as a kinetic mindfulness training that allowed for the practice of acceptance and reappraisal when, for example, a coloring mistake happened. With this in mind, Anima is a brain-computer interface that generates an adaptive color palette to foster awareness on one's mental states, and it is tailored to one's experience and interests as an aid to augment mandala coloring.

### Theoretical Framework

It has been shown that mindfulness practice and the development of mindfulness expertise is closely linked to increased awareness of the body and its sensations [28-31], which has been found to be beneficial for mental well-being in people with depression [1,5,32]. Despite the broad range of practices to train mindfulness, most interactive systems have focused on static guided meditation [33-35]. Our work builds on the practice of mandala coloring [21,36-38] as an alternative, less-explored approach to supporting focused-attention mindfulness training [39]. Originated in Buddhist traditions as a meditation aid, mandalas are a type of sacred geometry that represents harmony, wholeness, and the self [15]. Always starting from an epicenter, mandalas grow in concentric structures consisting of circles and layers that represent different aspects of the Tibetan Buddhist universe. Mandalas were brought to Western traditions by Carl Jung, who was the first to use the mandala as a therapeutic tool [14]. He found that the drawing of a mandala had a calming and healing effect on its creator by eliciting structure within the person's thoughts and ultimately creating a meditative state [40]. Ever since, mandalas have been used in art therapy to facilitate the emergence of inner experiences and feelings, which are expressed both consciously and unconsciously through art materials and the use of colors [21,38,41].

During the practice of mandala coloring, individuals need to focus on the coloring process, as the complex design provided by the mandalas requires a high concentration level. Small areas have to be colored with small and conscious movements [9,31,42], which in turn facilitates grounding in the present moment [42,43]. In contrast to static practices, such as sitting meditation, traditional movement-based mindfulness practices tend to rely on physical tools to restrict one's motion, such as copper funnels for sand mandalas [44], Baoding balls [45], or prayer wheels [46]. These tools are generally used as aids for grounding in the present moment while engaging in controlled, slow, and continuous movements [45]. Some of these traditional meditation artifacts have also influenced the design of mindfulness technologies, such as the Spheres of Wellbeing [47] or the Channel of Mindfulness [48]. In the case of mandala coloring, the tools that facilitate the mindfulness training would be the art materials used for coloring the geometry. Despite the increasing human-computer interaction and psychological interest in the role of art or craft materials [24,49-51] and technology for well-being [35,52-56], there is still a lack of systems that use art materials as active interaction cues. Further, designs inspired by movement-based mindfulness practices such as mandala coloring are still limited despite their potential to offer distinctive interactions for fostering the experience of an embodied self [28,33,36]. 

Recently, brain-computer interfaces (BCIs) have also been used to support the self-regulation of attention during mindfulness practices. For instance, MeditAid is an interactive system that uses neurofeedback during mindfulness sitting meditation to support the self-regulation of attention [57]. In this case, adaptive aural entrainment is controlled by the user's brain activity and their mindfulness state. Similarly, PsychicVR introduced an element of playfulness to the experience of sitting meditation, as it allows users to interact with the virtual environment [58]. Another example is Inner Garden, in which one's internal state is projected in a sand terrain that can be modified by shaping the sand [59]. These are some key illustrations of how BCIs have been used to differently augment mindfulness practices to enhance self-regulation processes. We argue that there is a less-explored design space, in which BCIs could be used to also augment movement-based mindfulness practices such as mandala coloring to foster mental well-being.

### Research Questions and Aims

The main objective of this research is to evaluate the impact of a novel mindfulness-based interactive technology (ie, Anima) on the mental well-being of people who have experienced depression.

The main research questions we seek to answer are the following: (1) To what extent can the materialization of brain activity using Anima facilitate the training of a mindfulness state for people who have depression? (2) To what extent does exposure to the training program positively influence the training of acceptance, self-awareness, and regulation of attention and emotions? (3) Does the use of Anima decrease depressive symptoms and increase mental well-being for people who have experienced depression?

The primary outcome is increased mental well-being after the study in comparison to baseline. The secondary outcomes are improved acceptance, self-awareness, and self-regulation of attention and emotional strategies and increased trait mindfulness at the end of the study.

### Anima: A Brain-Computer Interface for Mandala Coloring With a Generative Color Palette

According to Jung, the psychotherapist that introduced mandala coloring to Western culture, the anima represents the inner personality, which allows the individual to bring attention toward unconscious parts of the self [55]. Previous research has shown that colors play an important role in mandala-coloring practices for well-being [24,38]. In this context, colors are used to better understand one's affective states while coloring the mandala by materializing current emotions onto colors or using colors to achieve the desired state. Furthermore, the practice of mandala coloring has been widely used in spiritual and mental well-being practices to facilitate the training of mindfulness [24,38]. Our Anima prototype aims to bring the attention inwards by materializing intangible processes (ie, mindfulness states) to facilitate the monitoring of mindfulness practice, such as mandala coloring. Building on work showing that mandala coloring fosters nonjudgmental focused attention [33,60], we sensitively designed Anima to augment the practice of mandala coloring by giving access to colors that represent one's mindfulness states in real time. The design was also inspired by traditional coloring and its interaction with the materials, which are placed within reach, there when needed, yet peripheral.

**Figure 1:**
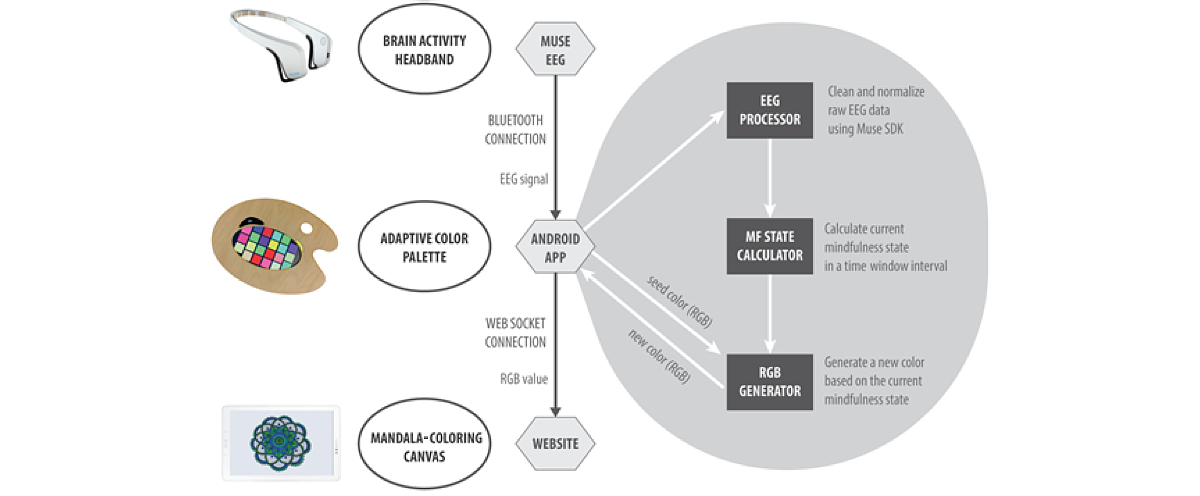
This diagram shows an overview of the system by describing the 3 components of Anima (ie, brain activity headband, adaptive color palette, and mandala-coloring canvas) and their integration. EEG: electroencephalogram; MF: mindfulness; RGB: red, green, and blue; SDK: software development kit.

Anima consists of 3 main components that have been carefully designed to fulfill a specific goal during its use (Figure 1): a brain activity headband, an adaptive color palette, and a mandala-coloring canvas. First, a brain activity headband is used to unobtrusively sense the electroencephalography (EEG) data, from which the mindfulness states of the person coloring the mandala are extracted. We will use Muse (Interaxon Inc) [61], a commercial unobtrusive brain activity headband that has been shown to provide valid and reliable measurements of event-related brain potentials in real time [62,63]. Previous work has also linked each of these brain waves with specific mental states [64], particularly during mindfulness training, from which mindfulness states can be clearly identified [13,65].

Second, the adaptive color palette is used as a peripheral interface to monitor the practice, as it provides new colors that are generated based on the current mindfulness state. The color palette is a hybrid object consisting of a tablet enclosed in a bespoke, wooden painter palette that adaptively provides a generative set of 22 colors from the user’s brain activity via an Android app we developed. The palette aims to subtly reflect the mental states involved in the unfolding mandala-coloring practice and to explore the ways that such materializations of mental states support mindfulness practice. When a color is selected from Anima’s generative palette (by tapping it), the canvas automatically loads it to color the digital mandala.

Finally, the canvas aims to recreate the traditional practice of mandala coloring to train focused attention. The canvas used to color the mandala consists of a tablet that displays a mandala from a website we developed and can be colored using a stylus. The color selected from the palette's Android app is automatically sent to the mandala canvas using web sockets, and it becomes available for coloring immediately.

## Methods

### Single-Case Experimental Design

In this study, we will follow the well-established methodology of a single-case experimental design (SCED). A SCED is an experimental research design in which an individual case serves as its own control, and the dependent variable measured is analyzed for each individual case and is not averaged across groups or across participants. This methodology emphasizes intensive repeated observations of a particular participant to demonstrate precise control over targeted behavior and includes a family of methods in which each participant serves as his or her own control [66]. There is an assortment of single-case designs. Dallery et al [67] discussed the purpose of each design as well as the similarities and differences between designs to evaluate novel technology-based health interventions. Following their assessment, this study will follow a combined approach of the multiple-baseline design and changing criterion design.

The multiple-baseline experimental design is a SCED in which a treatment is successively administered over time to different participants for different behaviors or in different settings. That is, in multiple-baseline designs, multiple AB data series are compared, and the introduction of the intervention is staggered across time. Comparisons are made both between and within data series. Adding phase repetitions increases the power of the statistical test, similar to adding participants in a traditional group design [68]. The number and timing of the repetitions can vary depending on the outcomes of the intervention. Among the characteristics of this design, effect replication across series is regarded as the characteristic with the greatest potential for enhancing internal and statistical conclusion validity.

The changing criterion design is a SCED in which a baseline phase is followed by successive treatment phases in which some criterion or target level of behavior is changed from one treatment phase to the next. The participant must meet the criterion of the treatment phase before the next treatment phase is administered. Thus, the changing criterion design is used to determine the effects of an independent variable when the final version of the target behavior cannot be emitted initially. Experimental control is demonstrated by the repeated changes in the dependent measure as the criterion is changed [69]. The steps in the changing criterion design must be large enough to clearly show the effects of the independent variable but not so large that the participant cannot meet the changed criterion. The critical element of changing criterion designs is the systematic introduction of a criterion level of performance over successive phases so that the behavior is essentially shaped into a final level, with each change in behavior occurring concurrently with the change in criterion. Experimental control is established by the simultaneous co-occurrence of both.

To sum up, the flexibility of SCED allows for greater freedom to ask innovative questions about novel treatments and has been widely used as an initial research design for testing innovative research in, for example, behavioral sciences [65] or novel technologies for health [67]. This methodology does not need a control group, as each participant acts as control during the baseline [68,70]. Although SCED is typically associated with low population validity, which is a subcategory of external validity, the external validity can be strengthened by generalizing across behaviors, participants, and settings [68,70].

### Study Setting

**Figure 2:**
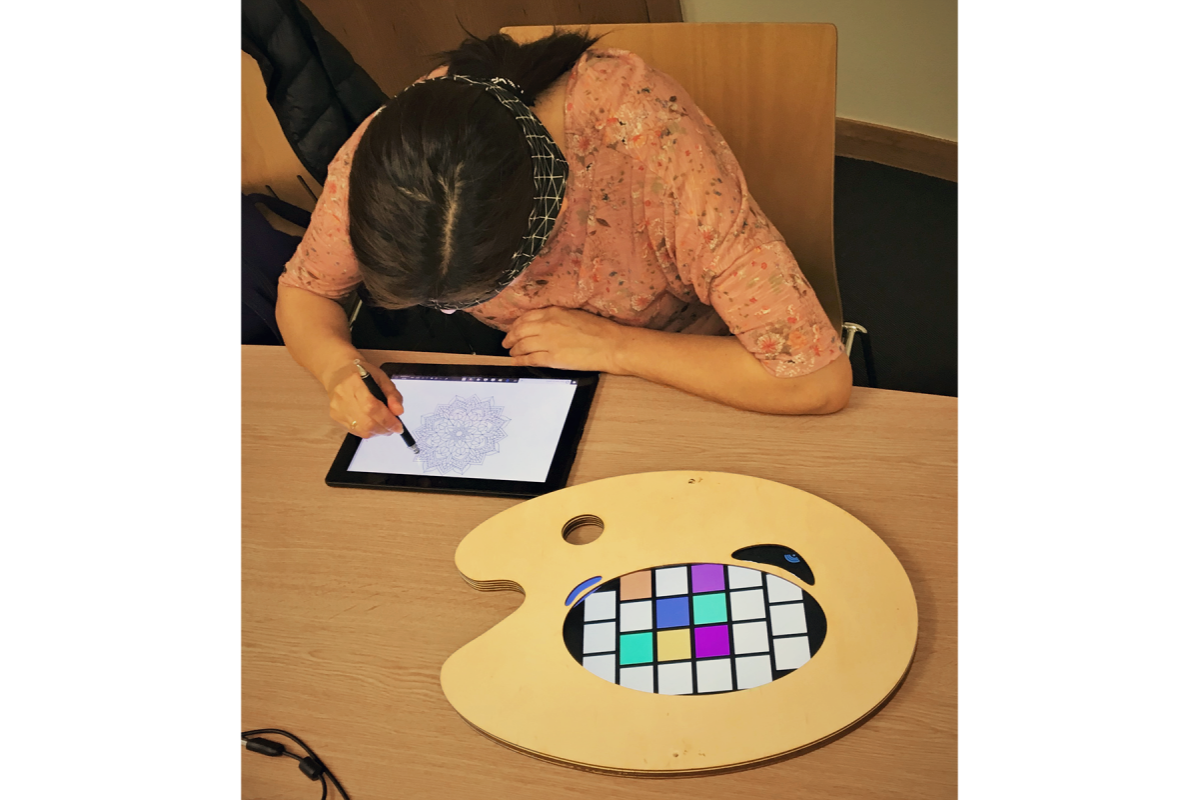
A person using Anima. While wearing the electroencephalography headband, colors based on the current mental states are generated on the wooden palette and can be used to color the digital mandala.

This training intervention will be carried out in the homes of eligible consenting participants. Participants will be asked to color a mandala using Anima (Figure 2) a total of 3 times a week, as described below, in their preferred quiet space in their house and during the evening if possible (ie, after work or other daily routines).

### Measures

Following previous studies evaluating the effect of mindfulness-based programs for health in general and depression in particular [71] (ie, MBAT [12,72], mindfulness-based cognitive therapy [7,73], and mindfulness-based stress reduction [74]), several instruments have been chosen to assess the dependent variables from the research questions.

Mindfulness

For state mindfulness [75], the Toronto Mindfulness Scale (TMS) was designed to assess mindfulness as a “quality maintained when attention is intentionally cultivated with an open, non-judgmental orientation to experience” [76]. The original TMS measures mindfulness as a state-like quality and not as a trait. The administration of the TMS requires that a brief mindfulness exercise precede self-administration of the instrument, and the TMS items assess the quality of that experience. The TMS is composed of 2 subscales, curiosity and decentering, and a total TMS score is not reported. Exploratory factor analysis suggested a 2-factor structure for the TMS, and this was supported by confirmatory factor analyses. The TMS has evidence of internal consistency, with the Cronbach α ranging from .86 to .91 and a Cronbach α of .85 and .87 for curiosity and decentering, respectively. Correlations for the decentering subscale with most of the other measures of mindfulness (*r*=0.20 to 0.74) were stronger than the correlations between the curiosity subscale and these measures (*r*=0.10 to 0.54) [77]. Curiosity and decentering were positively correlated with absorption, awareness of surroundings, reflective self-awareness, and psychological mindedness. As hypothesized, only curiosity was correlated with awareness of internal states and self-consciousness (*r*=0.41 and *r*=0.31, respectively), and only decentering was correlated with openness and cognitive failures (*r*=0.23 and *r*=–0.16). Decentering is posited to be a major outcome of mindfulness-based cognitive therapy and a mechanism that enables patients to be resilient to depressive thoughts, and patients with depression have lower levels of decentering compared with healthy controls [78].

For trait mindfulness [75], the Mindful Attention Awareness Scale (MAAS) was created to specifically capture attention and awareness in daily life [32]. It is a 15-item scale designed to assess a core characteristic of dispositional mindfulness, that is, open or receptive awareness of and attention to the present moment’s experiences. The scale shows strong psychometric properties (Cronbach α=.89) and has been validated with college students [79-81] and community [32] and cancer [82] patient samples.

Research has shown that the MAAS taps into a unique quality of consciousness that is related to and predictive of a variety of self-regulation and well-being constructs. It has also been found that the greater the change in mindfulness, the greater the reduction in depressed mood and the extent to which participants deal with difficulties through rumination and avoidance [83,84].

#### Depression Symptoms

The Beck Depression Inventory second edition (BDI-II) is a 21-item scale and one of the most widely used self-report measures of depression [85], with well-established psychometric properties (Cronbach α ranging from .83 to .96) [86].

#### Mental Well-Being

The Warwick-Edinburgh Mental Well-being Scale (WEMWBS) [87] is designed to capture a broad conception of well-being, including affective-emotional aspects, cognitive-evaluative dimensions, and psychological functioning. The scale consists of 14 items, each answered on a 5-point scale ranging from “none of the time” (1) to “all of the time” (4), and it is scored by summing all the items into a total well-being score (range of 14-70). The total score is the summation of all the items, with higher scores indicating greater well-being. The WEMWBS was assessed in the United Kingdom with 9 focus groups, one with mental health service users [88]. The Cronbach α is .91 for this scale.

#### Acceptance and Reflection

Private self-consciousness and the subordinate constructs of self-reflection and insight are key factors in the self-regulatory process underpinning the creation of behavior change in both clinical and nonclinical environments, and they can be assessed with the Self-Reflection and Insight Scale (SRIS) [89]. The SRIS self-reflection factor analysis correlated positively with anxiety and stress but not with depression and alexithymia, while the insight factor analysis was negatively correlated with depression, anxiety, stress, and alexithymia and positively correlated with cognitive flexibility and self-regulation. The coefficient α was .91 for the self-reflection scale and .87 for the insight scale.

#### Emotional State

The Self-Assessment Manikin (SAM) is a widely used nonverbal pictorial assessment technique used to obtain self-assessments of emotional state on the dimensions of affective valence, arousal, and dominance [90]. Each dimension is represented by one item that shows a picture of a manikin in 5 grades. Valence is operationalized by a manikin showing a negative or positive affective state, arousal is operationalized by a manikin being more or less energetic, and dominance is operationalized by showing a rather small (feeling of less dominance) or large manikin (feeling of much dominance). Despite the small item number, several studies indicate sufficient reliability of the SAM [60,91].

#### User Experience

User experience will be measured indirectly by how often participants use the prototype and directly during the interviews and using the User Experience Questionnaire (UEQ) after each session [92]. The average Cronbach α value for the English version of the UEQ is .79, which makes the reliability of the questionnaire sufficiently high. This measure has been evaluated in different scenarios [93], and a benchmark has been developed to facilitate the interpretation of user experience evaluations using UEQ [94]. In this study, we will use the short version (UEQ-S) [95], which has Cronbach α values between .81 and .85, as filling out the UEQ takes between 3 and 5 minutes, which might be too long to do after each session, deteriorating user experience.

### Data Collection

**Figure 3:**
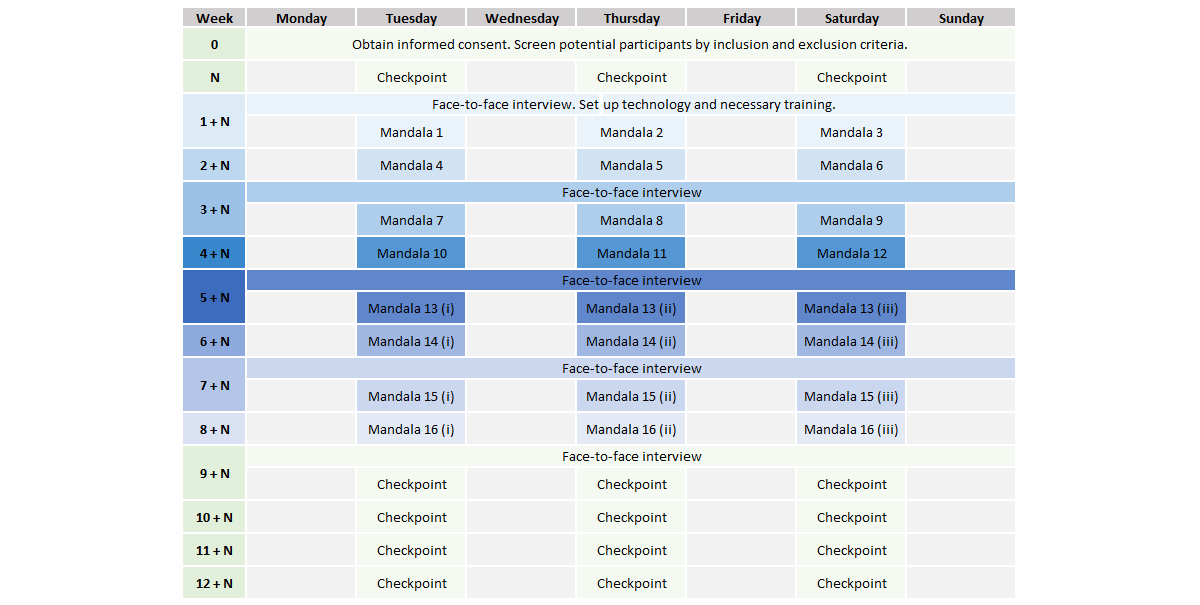
Timeline of the protocol, starting with N weeks of baseline per participant, followed by 8 weeks of intervention and 4 weeks of postintervention monitoring.

The study will have 3 phases (as shown in Figure 3): baseline, intervention, and monitoring. Following the multiple-baseline design, the duration of the baseline will vary depending on the participant, but the intervention phase will always last 8 weeks, and the monitoring phase will be 4 weeks. During the baseline and monitoring phases, participants will be asked to complete the trait measurements of trait mindfulness (ie, MAAS), depression symptoms (ie, BDI-II), mental well-being (ie, WEMWBS), and acceptance and reflection (ie, SRIS). These questionnaires will be filled in 3 times a week, and the total time expected for completing them all is about 34 minutes.

**Figure 4:**
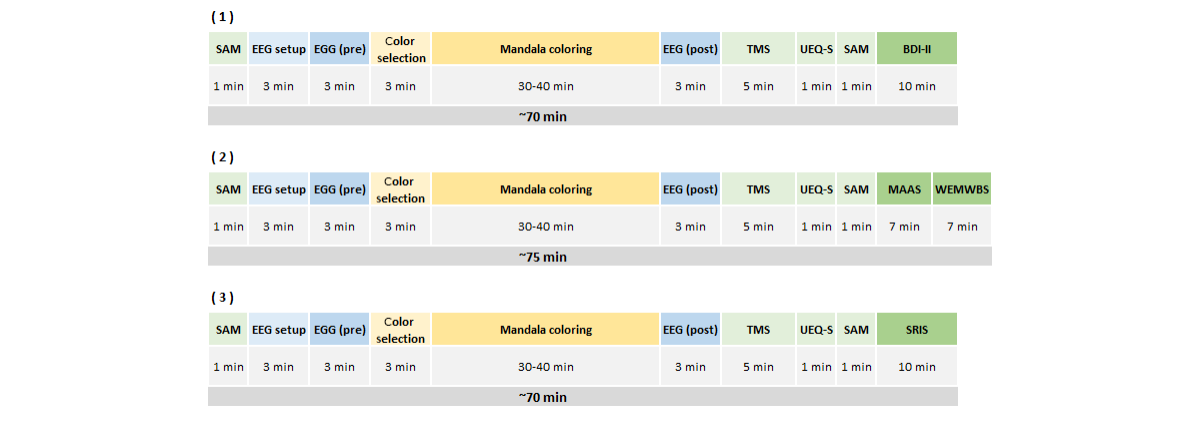
Triweekly mandala-coloring sessions during the intervention phase (in blue in Figure 3). To evaluate the effect of mandala coloring, data collection in each session will include pre- and post-EEG and measurements of emotional state (ie, SAM), mindfulness state (ie, TMS), and user experience (ie, UEQ-S). Trait measurements will be collected once a week. Hence, questionnaires will be distributed during the 3 sessions: BDI-II for depression symptoms in the first session, MAAS for trait mindfulness and WEMWBS for mental well-being in the second session, and SRIS for acceptance and reflection in the third session. BDI-II: Beck Depression Inventory second edition; EEG: electroencephalography; MAAS: Mindful Attention Awareness Scale; SAM: Self-Assessment Manikin; SRIS: Self-Reflection and Insight Scale; TMS: Toronto Mindfulness Scale; UEQ-S: User Experience Questionnaire short version; WEMWBS: Warwick-Edinburgh Mental Well-being Scale.

The intervention phase will last 8 weeks for all participants, starting at different points in time. Every week, they will be asked to practice mandala coloring using Anima 3 times. Each session will last between 70 and 75 minutes, as shown in Figure 4, and is divided into 4 stages: premeasurements and postmeasurements (marked in pale green), EEG data collection (marked in blue), and mandala coloring (marked in yellow). Instruments measuring trait will be completed once a week and distributed during the 3 weekly sessions (marked in darker green). All data will be collected using digital versions of each questionnaire on the tablet used as a canvas for mandala coloring.

Further, a short face-to-face semistructured interview will take place every 2 weeks with each participant to check that the technology is working and to gather qualitative data on their experience, both for the evaluation of user experience with Anima and for their mandala-coloring practice evolution.

### Data Analysis

The most common method of data analysis in SCEDs consists of conducting a visual analysis to determine intervention effects, as long as the baseline phase has been stable [68,70]. In this case, the stability of a measure is assessed by the consistency in the pattern of change in a dependent measure in each phase of a design. The more stable or consistent changes in a dependent measure are in each phase, the higher the internal validity of the research design. Furthermore, a measure can have a change in level or a change in trend, and the larger the magnitude of change (ie, size of the change in a dependent measure observed between phases of design), the greater the internal validity of the research design.

Although there are no specific guidelines for using statistical methods for analyzing SCED data, repeated measurements have been commonly used to evaluate the autocorrelation of sequential observations of the data. However, because of the nature of the SCED method, missing data can occur. Therefore, multilevel modeling and autoregressive moving average methods can be used to overcome these challenges.

## Results

### Ethics Approval

This study is currently in the process of being submitted to the National Health Service (NHS) to be reviewed by a research ethics committee (Integrated Research Application System number: 262687). Given the current situation and the NHS dealing with a global pandemic, as of summer 2020, we understand that this process may be delayed.

We now detail the sample and recruitment process for the study.

### Inclusion Criteria

All adults in the community who (1) are aged between 18 and 60 years, (2) have been diagnosed with mild to moderate depression in the past, (3) have finished treatment within the last year, and (4) are not currently being treated or on a waiting list for psychotherapy for any kind of mental health problem will be initially selected for the study. Further, in order to be included in the study, people will need to (1) show readiness to change, (2) show willingness to engage in self-care, (3) have an interest in interactive mindfulness practices, (4) have internet at home, and (5) have basic knowledge of how to use interactive technology (eg, regular usage of a smartphone, knowing how to connect two devices using Bluetooth).

### Exclusion Criteria

People with (1) motor impairments in the upper part of the body; (2) a major depressive disorder, bipolar disorder, or psychotic disorder based on the Diagnostic and Statistical Manual of Mental Disorders fifth edition criteria; (3) suicidal risk; or (4) a history of a major depressive disorder in the past 6 months according to Kupfer's model [96] will be excluded. It is also known that medication, drugs, and alcohol can highly affect brain activity [97]. Therefore, people with signs of alcohol misuse (ie, drinking more than 14 units a week) [64] and people undergoing a long-term medication treatment will be excluded. Finally, people who have actively engaged in mindfulness practices for the past year (ie, any type of meditation, yoga, tai chi, or qigong) or who score higher than 4 in the MAAS will be excluded from the study, as the number of years of meditation practice is positively related to the MAAS [32]. Likewise, people who have colored mandalas or adult coloring books more than once a week for the past 6 months will be excluded.

### Sampling

The method followed in this study is the well-established purposeful sampling method [98], which involves identifying and selecting individuals from a specific population group. In our case, this is people who have recovered from a depressive episode recently and have an interest in mindfulness (detailed description in “Inclusion Criteria” section).

Single-case experimental designs emphasize intensive repeated observations of a particular subject to demonstrate precise control over the targeted behavior [70]. Therefore, these designs usually select a limited number of individuals and collect a considerable amount of data per participant [67]. Based on previous work following SCED methodology [70], the estimated sample size for this study is 15 people.

### Recruitment

Participants of this study will be recruited through the Lancashire Care NHS Foundation Trust and will be able to withdraw at any time without justification. This provider will pass the invitation on to eligible residents so they can consider whether they would like to release their contact details to the research group. This study will only include participants who can provide their own informed consent. The service provider handing on the invitation will know whether the person can provide his or her own consent to participate as part of their service agreement with the resident.

## Discussion

This study follows the ethical guidelines and requirements by the European Union, Lancaster University, and the NHS. In terms of data collection and protection, Lancaster University will be the data controller for any personal information collected as part of this study under the General Data Protection Regulation. Further information about how Lancaster University processes personal data for research purposes and about individual data rights can be found on their webpage [99].

This protocol has been designed alongside a clinical psychologist with expertise in biofeedback from the AffecTech consortium. It was later iterated with the study support service from the National Institute for Health Research Clinical Research Network in the North West. The technology used in this study, Anima, has already been evaluated with the general population in 2 different settings: a public engagement event with mental health professionals in Lancashire and a workshop with people with experience coloring mandalas for mindfulness training and mental well-being (ie, they had been coloring mandalas at least monthly for the last year).

## Appendix

Multimedia Appendix 1 Peer-review reports (1).

Multimedia Appendix 2 Peer-review reports (2).

## Abbreviations

BCI: brain-computer interface
BDI-II: Beck Depression Inventory second edition
EEG: electroencephalography
MAAS: Mindful Attention Awareness Scale
MBAT: mindfulness-based art therapy
NHS: National Health Service
SAM: Self-Assessment Manikin
SCED: single-case experimental design
SRIS: Self-Reflection and Insight Scale
TMS: Toronto Mindfulness Scale
UEQ: User Experience Questionnaire
WEMWBS: Warwick-Edinburgh Mental Well-being Scale
