# Supplementary material for: A Mindfulness-Based Brain-Computer Interface to Augment Mandala Coloring for Depression: Protocol for a Single-Case Experimental Design
Source: JMIR Res Protoc. 2021 Jan 18;10(1):e20819. doi: 10.2196/20819 (PMC7850910; doi:10.2196/20819)
Supplement: Multimedia Appendix 1 [file resprot_v10i1e20819_app1.pdf]

The Anima study protocol presents a mindful-based self-administered training for the enhancement of mental wellbeing in people who have suffered from depression.

The study protocol lasts 8 weeks and will include 15 participants.

**Importance and potential impact of the proposed research**

- The research identifies a need that could be managed with the tools proposed, i.e. the possibility to self-enhance mental wellbeing using new technologies Is the research question posed the most important question? If not why not? How could the question be improved?
- The impact of the study could be related to the follow-up of patients having suffered from depression
- The impact is more related to applied scientific knowledge
- The research protocol and the tools used for self-training is quite innovative in the scientific community
- The proposal make a clear case for the research proposed with reference to the current evidence base, but more information are needed in some points (see section 1.5)

**Patient and public involvement**

- Not specified

**Research outputs**

- No mention has been made about the intellectual properties and the future exploitation development

**Key Strengths (1.2)**

- The key strengths of the proposal are related to the fact that the proposed set up is easy enough to be self-managed, and if efficient, could offer great opportunities for a wide range of people, not only those having suffered from depression, but ideally all the people managing stressful situations or anxiety. The possibility of self-training is a core added value since it allows an autonomous administration and make its use more feasible also for people that for different reasons, do not have access to public mental health system.

**Key Weaknesses (1.3)**

- There are only few points to address to strengthen the proposal, and they listed in section 1.5

You may wish to consider the following questions when preparing your response in sections 1.2 and 1.3:

**Consideration of methods and scientific quality**

- The research protocol is feasible, and the method quite robust
- The protocol is designed as to provide answers to the research questions
- The plans for data collection and analysis  are sufficiently detailed
- The sample size must be justified (see comments below)
- The interventions adequately described and appropriate, but Figure 1 could be improved
- The effects, measures and information sought are appropriate and relevant to the study's purpose
- The duration of follow up is appropriate for a mid-term efficacy verification
- No mention of the analyses planned has been made.

**Research team, resources and research management**

- The research team is not described
- Have the research team stated that this protocol was built upon previous studies on Healthy subjects, ma references to those studies are missing (at the end of page 1).
- The applicants demonstrate an adequate track record appropriate for the reaserch plan they aim to pursue
- The chance of recruiting the required number of participants is realistic in a quite long period of time. No mention about the schedule planned by researchers; the willingness to participate can be predicted as high, since the procedure looks pleasant and not too much invasive participation
- No information about the costs are provided

**Ethics and governance**

- The proposed research is ethically sound and will comply with the GDPR regulation. No information is provided about the informed consent obtained from participants (i.e. it should be clearly stated that no clinical information can be drawn from the use of the EEG) device

**Plain English summary (1.4)**

The plain English summary has not been submitted.

**Questions for applicants (1.5)**

I suggest the following actions to improve the proposal:

- Make it clearer whether there are previous studies indicating M-BAT efficacy

- The background and the theoretical framework sections looks overlapping in many points. Just make sure not to say the same thing twice.
- Figure 1 is unclear to a reader: the interviews are conducted every day?
- Please better specify how the colours are related to the EEG recordings: which rule or algorithm has been used?
- Is there the possibility to monitor how long the training is actually performed bìy each participant?
- Inclusion criteria: please provide justification for the inclusion criteria b to d; why not using patients who are being depressed?
- Please justify how sample size has been calculated
- Please describe how data will be analysed in relation to the research design selected. In addition it is not clear to me how the Changing criterion design applies to these specific data.

**Section 2: Score and confidential comments**

No further comments

**Comments field (2.1)**

**NA**

**Summary score (2.2)**

Please provide a summary score that reflects your overall assessment of the proposed research

Scoring can often be challenging, particularly as you may be keen to see research into an important area go forward. It may be worth considering the following table when deciding on the score to give the proposal. Your score should reflect your overall assessment of the proposal. For more information about the funding process please see Appendix A.

| Score | Description of application | Suggested outcome | What this means from your perspective, including considerations around the delivery, PPI, conduct and dissemination of the proposed research. |
|---|---|---|---|
| 6 | Excellent | Proposed research can be funded as it stands | I have no concerns about this proposal. |
| 5 | Good | Proposed research can be funded with minor changes | The concerns that I have about this proposal could be easily corrected. |
| 4 | Good potential | There is much merit in this proposal, but it could be funded, perhaps after | The concerns that I have about this proposal could be corrected. |

| | | | |
|---|---|---|---|
| | | resubmission, with additional external support | |
| 3 | Some merits | There are significant weaknesses in this proposal, but these could in principle be addressed. | I have significant concerns but it may be possible for these to be addressed, although it would not be straightforward to do so. |
| 2 | Poor | Weak proposal | I have very strong concerns, which would be difficult to address, but the proposal does have some merit. |
| 1 | Extremely poor | Unsupportable proposal | I have very strong concerns that would be extremely difficult to address. |
