# Supplementary material for: A Mindfulness-Based Brain-Computer Interface to Augment Mandala Coloring for Depression: Protocol for a Single-Case Experimental Design
Source: JMIR Res Protoc. 2021 Jan 18;10(1):e20819. doi: 10.2196/20819 (PMC7850910; doi:10.2196/20819)
Supplement: Multimedia Appendix 2 [file resprot_v10i1e20819_app2.pdf]

NIHR | National Institute for Health Research

The review task

Section 1: Review Questions

The following points are provided as a guide towards what you may wish to consider when answering the questions; however, we would be grateful for any feedback or other points that you would like to raise as a result of your specialised knowledge, skills and experience.

Your responses to the questions in section 1 are normally shared anonymously with applicants prior to consideration by the funding committee. Applicants are then given the opportunity to respond to the issues raised by reviewers prior to a final committee decision.

If you have any questions or issues you think the applicants should respond to prior to consideration by the funding committee then please raise these in question 1.5. The text boxes will expand to accommodate your comments.

Issues for consideration by the Committee (1.1)

This section can be presented as 'major' (those issues which could prevent the application form being funded) or 'minor' (issues which could be resolved).

You may wish to consider the questions below when preparing your response (where applicable).

Importance and potential impact of the proposed research

- Does the research meet an identified need (e.g. a gap in the evidence base)? Does it meet the requirements as specified in the commissioning brief/specification document?
- Is the research question posed the most important question? If not why not? How could the question be improved?
- What impact is this research likely to have on clinical practice, improving health services and/or public health?
- What impact is this research likely to have on scientific knowledge?
- Does this research 'overlap' with other similar research that you are aware of?
- Does the proposal make a clear case for the research proposed with reference to the current evidence base?

  The research plan meets an identified need, that is, to enhance mental wellbeing in people who have suffered from depression.

  The research question is relevant and if the objectives are met, this will be a relevant contribution to the literature.

Patient and public involvement

- Have patients, carers or members of the public been involved in the development of the research proposal? In what capacity?
- In what capacity will patients, carers or members of the public be involved in the implementation of the proposed research? Is this appropriate?
- Have appropriate resources been allocated to ensure effective patient and public involvement?

  There are people (former patients) involved in the development of the research proposal and this is appropriate, because, they are going to be the participants in this study.

Research outputs

- Is the proposed research based on Intellectual Property (IP) developed by others? Does the applicant have access to use the existing IP? Who will own any future IP?
- Have the team considered Intellectual Property (IP) being generated by this study? (e.g. new or improved checklist; patient data; software tool; tool kit for patient care; database design; medical device, pharmaceutical drug, or application; equipment design; new data of corporate significance)
- Has the project team identified any potential to exploit developments and with whom?
- Do you believe there to be any additional opportunities to exploit or further develop the outputs from the proposed research?
- Is a commercial partner involved in the study? Is there a likely need for collaboration agreements or sub-contracts?
- Are there any changes that should be made to the research to make it better for patients taking part in it?

This research project is being carried out within the framework of a Marie Curie grant and, as has been done in other centres that are part of this grant, it is hoped to identify potentials to exploit this development.

This reviewer is not aware of the presence of potential business partners in the study

Key Strengths (1.2)

- What are the key strengths of this proposal? Please give an overall assessment of the key strengths of the research proposal.

It is clearly multidisciplinary and proposes to use a technological device to improve the well-being of people who have suffered a clinical condition such as depression

Key Weaknesses (1.3)

- What are the key weaknesses of this proposal? Please identify and summarise any aspects of the proposal that you perceive to be a weakness.

In the proposal is indicated that this intervention will decrease the depressive symptoms of people who have suffered from depression. But, the clinical status of the participants should be indicated at the time the study begins. That is, the inclusion criteria should be clarified.

You may wish to consider the following questions when preparing your response in sections 1.2 and 1.3:

Consideration of methods and scientific quality

- Is the proposed research feasible? Yes
- Are the research methods robust? Yes
- Will the research methods suggested provide answers to the research question(s)? Yes
- Are the plans for data collection and analysis sufficiently detailed? The global explanation and the name of the third phase of the study is not clear. In the multiple baseline designs, it is necessary to carry out "monitoring" through all the phases of the study. Then, Figure 1 should be improved.
- Are any assumptions made? Are they reasonable? Yes
- Is the study design appropriate? Yes
- Are the research subjects and the methods of identifying them relevant and sufficient? Yes
- Are the interventions adequately described and appropriate? Yes
- Are the effects, measures or information sought appropriate and relevant to the study's purpose? Yes
- Are the outcomes and duration of follow up appropriate? Yes
- Do the planned analyses appear reasonable? Yes
- Where appropriate, is there a clear explanation and justification for the sample size and/or estimated recruitment rates? Yes
- Is the sample size based on a believable effect size? This is a single-case design
- Are there any changes that should be made to the research to make it better for patients taking part in it? No

Research team, resources and research management

- Does the research team have an appropriate mix of skills (i.e. researchers from different disciplines, managers and professionals, patients and/or public members)?
- Is the role of each collaborator/co-applicant clear?

- Have the research team sought representation from relevant professional groups or practitioners required to inform the study design and delivery?
- Do the applicants demonstrate an adequate track record appropriate to their proposed roles?
- If the study involves more than one organisation, are the relationships between the various organisations clearly defined?
- Are the resources requested appropriate for the scale and methods of the proposed study?
- Where appropriate a) is there a realistic chance of recruiting the required number of participants within the time stated and b) if you had the relevant condition, would you be willing to participate as a patient in the study?
- Does the proposal represent good value for money? Does the total funding required (research grant and NHS costs) represent good value for money?
- Are the applicant's estimates of the resource implications of the patient care costs (NHS Support and Treatment costs, including Excess Treatment costs) substantially over-estimated, substantially under-estimated or about right?

No problems regarding these questions

Ethics and governance

- Is the proposed research ethically sound?
- Are the study governance arrangements appropriate?
- Have the necessary approvals been obtained (or measures included to address this) from the relevant ethical and regulatory bodies?
- Have the applicants indicated that they are aware of the appropriate trial monitoring structures that need to be in place, and how they intend to to implement them? Eg Process for informed consent, a Trial Steering Committee (TSC), a Data monitoring and Ethics Committee (DMEC).

No problems regarding ethical issues

Plain English summary (1.4)

The plain English summary is intended for an interested audience, who are not necessarily specialists. The summary should be written at roughly the same level as an article in a newspaper. With this in mind, please comment on the following:

- Does the plain English summary give a clear explanation of the research?
- Does it help you carry out your review? If not, why not?
- Is the language used appropriate and clear? If not, where are there problems?
- Are scientific terms, abbreviations and jargon explained? If not, which terms need explanation?

- If this research is funded, the plain English summary will be published on a variety of websites without the rest of this application form. Could this plain English summary be used on its own to describe the proposed research? If not, what further information is needed?

Further information for researchers on how to write a plain English summary and what to include in a summary is available online at NIHR Make it clear.

The summary is adequate

Questions for applicants (1.5)

If you have any questions you would like to raise with the applicants prior to an assessment by the funding committee, please raise them in this section. These questions are sent anonymously to the applicant who is then given an opportunity to respond. The funding committee will see the applicant's response to these questions.

Section 2: Score and confidential comments

If you wish to raise any issues in confidence then you can do so in section 2.1 'Comments field'. In section 2.2 we ask you to provide a summary score. If you wish to provide a justification for your score then please do so in section 2.1.

Comments field (2.1)

Please use this section to raise any other comments you would like to make in confidence from the applicants.

This section should be used if you are concerned that making a comment may identify you to the applicants. It would be most useful if the majority of your comments were made in prior sections as your feedback may help applicants to improve their application. If you are unsure about this or have any queries then please contact us.

Summary score (2.2)

Please provide a summary score that reflects your overall assessment of the proposed research

Scoring can often be challenging, particularly as you may be keen to see research into an important area go forward. It may be worth considering the following table when deciding on the score to give the proposal. Your score should reflect your overall assessment of the proposal. For more information about the funding process please see Appendix A.

Score	Description of application	Suggested outcome	What this means from your perspective, including considerations around the delivery, PPI, conduct and dissemination of the proposed research.

6	Excellent	Proposed research can be funded as it stands	I have no concerns about this proposal.
5	Good	Proposed research can be funded with minor changes	The concerns that I have about this proposal could be easily corrected.
4	Good potential	There is much merit in this proposal, but it could be funded, perhaps after resubmission, with additional external support	The concerns that I have about this proposal could be corrected.
3	Some merits	There are significant weaknesses in this proposal, but these could in principle be addressed.	I have significant concerns but it may be possible for these to be addressed, although it would not be straightforward to do so.
2	Poor	Weak proposal	I have very strong concerns, which would be difficult to address, but the proposal does have some merit.
1	Extremely poor	Unsupportable proposal	I have very strong concerns that would be extremely difficult to address.

In my opinion, the research proposal gets a score of 5.5. Between Good and Excellent. It would only be necessary to explain better the admission criteria of the participants and to slightly improve the plan for monitoring the participants.

Appendix A: The funding process

When considering stage 2 applications, NIHR committee members have the responsibility to take into account the views from all reviewers and other committee members to decide what research to recommend for funding.

Committee members are asked to score each application based on the view they have formed, and an average score is calculated for the proposal. The highest scoring applications will be recommended for funding. Those proposals that are deemed ‘important’ but have other issues, which may prevent them achieving a high score, can be designated as a ‘fund with change’. All proposals which are designated as a ‘fund with change’ are required to go through an iterative process to ensure that they have corrected all issues before they are taken further.

All funding committees include public members, and all stage 2 applications should also have an assessment by an appropriately experienced member of the public.

NIHR | National Institute for Health Research
